# Mucolipin-2 Cation Channel Increases Trafficking Efficiency of Endocytosed Viruses

**DOI:** 10.1128/mBio.02314-17

**Published:** 2018-01-30

**Authors:** Nicholas Rinkenberger, John W. Schoggins

**Affiliations:** a Department of Microbiology, University of Texas Southwestern Medical Center, Dallas, Texas, USA; Stony Brook University

**Keywords:** endocytosis, flavivirus, influenza, ion channels, vesicular trafficking, virus entry, virus-host interactions

## Abstract

Receptor-mediated endocytosis is a cellular process commonly hijacked by viruses to enter cells. The stages of entry are well described for certain viruses, but the host factors that mediate each step are less well characterized. We previously identified endosomal cation channel mucolipin-2 (MCOLN2) as a host factor that promotes viral infection. Here, we assign a role for MCOLN2 in modulating viral entry. We show that MCOLN2 specifically promotes viral vesicular trafficking and subsequent escape from endosomal compartments. This mechanism requires channel activity, occurs independently of antiviral signaling, and broadly applies to enveloped RNA viruses that require transport to late endosomes for infection, including influenza A virus, yellow fever virus, and Zika virus. We further identify a rare allelic variant of human *MCOLN2* that has a loss-of-function phenotype with respect to viral enhancement. These findings establish a mechanistic link between an endosomal cation channel and late stages of viral entry.

## IMPORTANCE

Viruses must co-opt cellular processes to complete their life cycle. To enter cells, viruses frequently take advantage of cellular receptor-mediated endocytosis pathways. A growing number of host proteins are implicated in these viral uptake pathways. Here, we describe a new role for the gated cation channel MCOLN2 in viral entry. This endosomal protein modulates viral entry by enhancing the efficiency of viral trafficking through the endosomal system. Thus, MCOLN2-mediated enhancement of infection may represent a key vulnerability in the viral life cycle that could be targeted for therapeutic intervention.

## INTRODUCTION

Animal viruses enter host cells by direct penetration at the plasma membrane or by endocytosis. Endocytic viruses rely on diverse uptake mechanisms, including but not limited to clathrin-mediated endocytosis, macropinocytosis, and caveolin/lipid raft-mediated uptake. A growing number of cellular host factors are involved in these diverse viral uptake pathways, including coat proteins (clathrin and caveolin), scission factors (dynamin 2), and regulatory and trafficking factors (Ras, RAC1, CDC42, phosphatidylinositol 3-kinase [PI3K], Rab GTPases, etc.) (1). Identifying new factors that regulate these viral entry processes is critical for understanding the complexities of the viral life cycle and for identifying key vulnerabilities in the infection process.

In recent screening efforts for interferon (IFN)-inducible factors that modulate viral infection, we found that mucolipin-2 (MCOLN2) enhanced the infectivity of diverse viruses, including yellow fever virus, dengue virus, influenza A virus, and equine arteritis virus (2, 3). MCOLN2 belongs to the transient receptor potential (TRP) protein superfamily, which consists of gated, tetrameric cation channels with diverse physiological functions, particularly in sensory signaling. These proteins share a conserved structure of six transmembrane helices with differing cytoplasmic oriented N- and C-terminal domains. Most TRP proteins are localized to the plasma membrane (4, 5). However, the transient receptor potential mucolipin-like (TRPML) family of proteins are localized predominantly to endosomes, where they have roles in vesicular trafficking, autophagy, and membrane fusion (6, 7).

The TRPML family contains three proteins, MCOLN1, MCOLN2, and MCOLN3. These TRPML family channels produce inwardly rectifying cation currents (8–10). Loss-of-function mutations in mucolipin-1 cause the human autosomal recessive disorder mucolipidosis type IV (ML-IV) (11, 12). This disorder is characterized by severe mental and psychomotor retardation, retinal degeneration, and hypotonia (13, 14). MCOLN1 is ubiquitously expressed, localizes primarily to late endosomes and lysosomes, and is known to play roles in lysosome maturation, lysosome fission, late endosome-lysosome fusion, autophagosome-lysosome fusion, lysosome expulsion, and iron release (10, 15–22). MCOLN1 also promotes autophagy proliferation via transcription factor EB (TFEB) activation (19, 23–25). Mutations in mouse *Mcoln3* cause the Varitint-Waddler phenotype, characterized by early onset hearing loss, vestibular defects, and pigmentation defects (26). MCOLN3 localizes to the plasma membrane, early endosomes, and to a lesser extent, late endosomes. Loss of MCOLN3 has been found to promote epidermal growth factor (EGF) degradation and delay autophagosome clearance (27, 28). Recently, a role for MCOLN3 in the expulsion of bacteria from bladder epithelium via a lysosomal expulsion pathway was identified (29).

By comparison, the function of MCOLN2 is poorly understood. MCOLN2 is thought to localize predominantly to recycling endosomes and have a role in an Arf6-associated recycling pathway (30, 31). MCOLN2 is expressed at low levels in most tissues with higher levels in the thymus and spleen (32). MCOLN1 and MCOLN3 have not been reported to be sensitive to IFN. However, MCOLN2 is an interferon-stimulated gene (ISG) shown to be induced in mouse macrophages in response to lipopolysaccharide (LPS) (31) as well as in chimpanzee peripheral blood mononuclear cells in response to type I IFN (33). The B cell transcription factor PAX5 has also been found to promote MCOLN2 expression (34). In a mouse model, *Mcoln2* knockout resulted in impaired chemokine secretion and reduced peripheral macrophage recruitment after bacterial challenge (31). Together, these findings implicate a role for MCOLN2 in immunity. However, the specific effects of MCOLN2 on viral infection are unknown.

Here, we characterize the mechanism by which MCOLN2 enhances viral infection. We found that MCOLN2 enhances viral entry. Specifically, MCOLN2 promotes trafficking of viruses from early to late endosomes resulting in increased release to the cytosol. This process is dependent on the channel activity of MCOLN2 and does not involve regulation of IFN signaling. Intriguingly, a rare genetic variant of *MCOLN2* fails to enhance viral infection in our cell culture model. Overall, our findings reveal a role for MCOLN2 as an endosomal host factor that modulates entry of a diverse group of endocytosed viruses.

## RESULTS

### MCOLN2 enhances infection of diverse RNA viruses.

**FIG 1:**
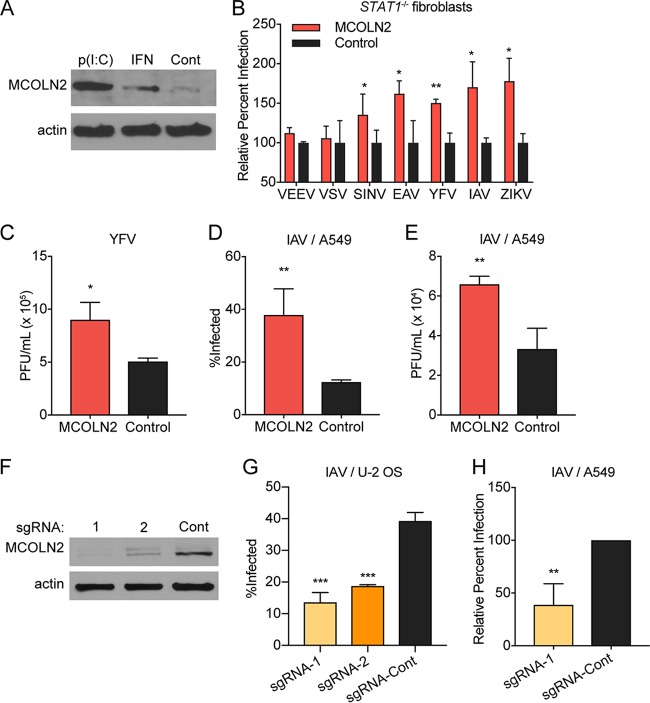
MCOLN2 enhances viral infection. (A) Western blot of lysates from THP-1 cells treated with 1,000 U IFN-α, 1 µg/ml poly(I·C), or PBS (negative control [Cont]) for 24 h. (B) *STAT1*^*−/−*^ fibroblasts were infected with the indicated viruses, and infectivity was quantified by flow cytometry. (C) Viral titers in supernatants of *STAT1*^*−/−*^ fibroblasts infected with YFV (MOI of 1) were determined by plaque assay in BHK-21J cells. (D and E) A549 cells were infected with IAV for 8 h, and infection was quantified by flow cytometry (D), or viral titers in supernatants were determined by plaque assay in MDCK cells (E). (F) MCOLN2 protein expression levels in U-2 OS cells targeted with the indicated CRISPR/Cas9 guides shown. sgRNA, single guide RNA. (G and H) CRISPR/Cas9-targeted U-2 OS (G) or A549 (H) cells infected with IAV at an MOI of 0.5 for 8 h. Infections were quantified by staining cells with anti-NP antibody and subsequent flow cytometry-based analysis. Values are means plus standard deviations (SD) (error bars) from three independent experiments performed in technical duplicate or triplicate. Values that are significantly different are indicated by asterisks as follows: *, *P* ≤ 0.1; **, *P* ≤ 0.01; ***, *P* ≤ 0.001.

To confirm that MCOLN2 is IFN inducible, we treated THP-1 monocytes with alpha interferon (IFN-α) or poly(I·C) and assessed MCOLN2 expression by Western blotting 24 h later (Fig. 1A). As previously reported, MCOLN2 is induced in response to type I IFN treatment (33). In previous screening efforts, we showed that MCOLN2 enhances infection of viruses from multiple families, including yellow fever virus (YFV) (*Flaviviridae*), influenza A virus (IAV) (*Orthomyxoviridae*), and equine arteritis virus (EAV) (*Arteriviridae*). However, MCOLN2 had no effect on Venezuelan equine encephalitis virus (VEEV) (*Togaviridae*), respiratory syncytial virus (RSV) (*Rhabdoviridae*), or vesicular stomatitis virus (VSV) (*Paramyxoviridae*) (2, 3). To confirm these results and extend our findings to Zika virus (ZIKV) (*Flaviviridae*), immortalized *STAT1*^*−/−*^ human skin-derived fibroblasts stably expressing MCOLN2 or a control vector were generated using a lentiviral expression cassette coexpressing tagged red fluorescent protein (TagRFP). Cell lines were infected at a low multiplicity of infection (MOI) with recombinant green fluorescent protein (GFP)-expressing reporter viruses (YFV, EAV, VEEV, Sindbis virus [SINV], and VSV) or nonreporter strains of IAV (WSN) and ZIKV (PRVABC59). After approximately one viral replication cycle, infectivity was assessed by quantitating the percentage of GFP-positive cells using flow cytometry. Alternatively, for IAV and ZIKV, we used virus-specific antibodies to quantify infectivity. Ectopically expressed MCOLN2 had similar effects on each of the previously studied viruses, as expected (Fig. 1B). We also found that MCOLN2 enhanced ZIKV infection by approximately 75% over control cells. Similarly, cells ectopically expressing MCOLN2 released nearly twice as much infectious virus when infected with a nonreporter YFV (Fig. 1C). To test whether the enhancing effect of MCOLN2 extends to other cellular backgrounds, we stably expressed MCOLN2 or an empty vector in human A549 lung adenocarcinoma cells and challenged cells with IAV. MCOLN2 expression enhanced both IAV infectivity (Fig. 1D) and infectious virus production (Fig. 1E).

To determine whether loss of MCOLN2 expression affects viral infection, MCOLN2 was knocked out of U-2 OS and A549 cells using the clustered regularly interspaced short palindromic repeat (CRISPR)/Cas9 system. Loss of expression in U-2 OS cells was confirmed by Western blotting (Fig. 1F). In both cell types, loss of MCOLN2 expression caused a significant reduction in viral infection (Fig. 1G and H). Together, these data indicate that endogenous MCOLN2 is required for optimal infection in diverse cell types and that infection can be enhanced by ectopic MCOLN2 expression.

### MCOLN2-mediated enhancement is not dependent on the IFN response.

**FIG 2:**
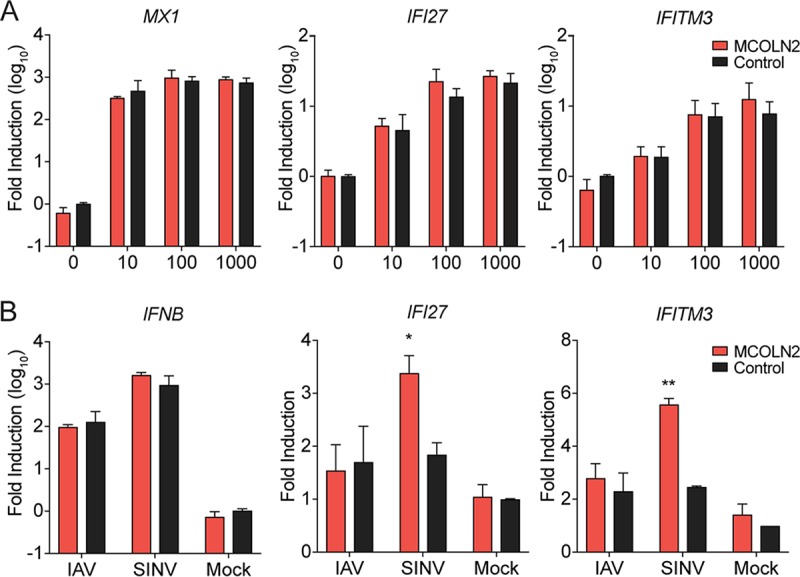
MCOLN2-mediated enhancement of viral infection is not dependent on the IFN response. (A and B) A549 cells were treated with the indicated doses of IFN-α for 24 h (A) or infected with IAV or SINV-GFP at an MOI of 1 for 8 h (B). ISG induction was quantified by RT-qPCR. Values are means plus SD (error bars) from three independent experiments performed in technical duplicate or triplicate. Values that are significantly different are indicated by asterisks as follows: *, *P* ≤ 0.1; **, *P* ≤ 0.01.

Since the MCOLN2 enhancing effects occurred in *STAT1*^−/−^ cells, we suspected that its effects were direct and not linked to negative regulation of antiviral signaling. However, MCOLN1 has been shown to modulate viral pathogen-associated molecular pattern (PAMP) recognition during Toll-like receptor (TLR) signaling (35). We therefore tested whether MCOLN2 modulates antiviral signaling in IFN-responsive A549 cells. Cells ectopically expressing MCOLN2 or control cells were treated with IFN-α and assessed for antiviral ISG induction by reverse transcription-PCR (RT-PCR). By using various doses of IFN, we found no differences in *MX1*, *IFITM3*, or *IFI27* induction between the two cell types (Fig. 2A). We next monitored *IFNB1* and ISG induction in MCOLN2-expressing cells or control cells infected with either IAV or Sindbis virus (SINV) (Fig. 2B). IAV infection is enhanced by MCOLN2 (Fig. 1A) but blocks IFN signaling by multiple mechanisms (36). SINV infection is similarly affected by MCOLN2 (Fig. 1A). MCOLN2 promoted ISG but not *IFNB1* expression in response to SINV, despite having a net positive effect on viral infection. Induction of *IFNB1* and ISGs in response to IAV was not affected by ectopic MCOLN2 expression (Fig. 2B). Combined with our original discovery of an MCOLN2 phenotype in a *STAT1*^−/−^ background (2), these data suggest that MCOLN2-mediated enhancement of viral infection is not linked to impairment of IFN or ISG induction.

### MCOLN2 channel activity is necessary for enhancement of viral infection.

**FIG 3:**
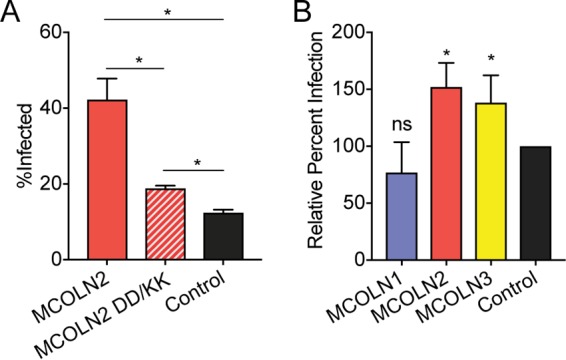
MCOLN2 enhancement of viral infection is channel dependent. (A and B) A549 cells were infected with IAV at an MOI of 0.2 for 8 h. Infections were quantified by staining cells with anti-NP antibody and subsequent flow cytometry-based analysis. Values are means plus SD (error bars) from three independent experiments performed in technical triplicate. Values that are significantly different (*P* ≤ 0.1) are indicated by a bar and asterisk. ns, not significantly different.

To determine whether MCOLN2 channel activity is important for its viral enhancing effect, we tested the infection phenotype of a well-characterized dominant-negative mutant, referred herein as MCOLN2-DD/KK, in which two conserved aspartates D463 and D464 are mutated to lysine. Homologous mutations in all MCOLN family members perturb the selectivity pore of the channel and have been found to prevent cation flow through these channels (8, 17, 27, 30). When stably expressed in A549 cells, the MCOLN2-DD/KK mutant was unable to enhance IAV infection (Fig. 3A), suggesting that channel activity is required for the viral phenotype. Since all members of the TRPML family localize to endosomal compartments, we examined whether any other members of this family enhanced viral infection (Fig. 3B). MCOLN3, but not MCOLN1, enhanced IAV infection when ectopically expressed.

### MCOLN2 enhances early, but not late, stages of the viral life cycle.

**FIG 4:**
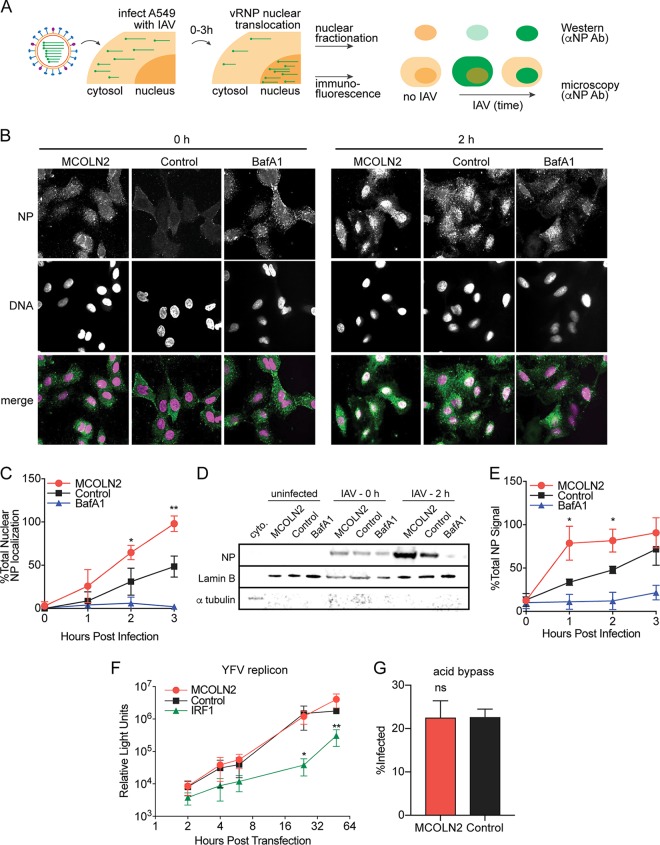
MCOLN2 specifically enhances viral entry. (A) Schematic diagram of experiments shown in panels B to E. (B to E) IAV (MOI of 20) was bound to A549 cells for 1 h at 4°C. The cells were rapidly shifted to 37°C in the presence or absence of 15 nM bafilomycin A1 (BafA1) for the indicated times. The cells were fixed and stained with anti-NP antibody (αNP Ab) (green), and nuclei were stained with Hoechst 33342 (blue). NP nuclear localization was assessed by confocal microscopy (B). NP nuclear localization was quantified with ImageJ (C). (D and E) Nuclear-cytoplasmic fractionation was conducted on infected cells (D). Nuclear viral NP content was determined by quantitative Western blotting (E). (F) Luciferase assay of lysates from *STAT1*^*−/−*^ fibroblast cell lines at the indicated time points after transfection with YFRP-Rluc subgenomic replicon RNA. (G) IAV (MOI of 10) was bound to A549 cells for 1 h at 4°C. The cells were rapidly shifted to 37°C in 50 mM acetic acid buffer (pH 5) for 5 min. The infection was continued for 8 h in pH 7 medium before harvest, staining with anti-NP antibody, and flow cytometry-based infection quantitation. Values are means ± SD (error bars) from three independent experiments. Values that are significantly different are indicated by asterisks as follows: *, *P* ≤ 0.1; **, *P* ≤ 0.01. ns, not significantly different.

We next sought to determine which steps of the viral replication cycle were affected by MCOLN2. To examine viral entry, we first focused on IAV. IAV virions enter cells through endocytosis, and the viral genome escapes into the cytosol after pH-dependent fusion of the viral envelope with the endosome. Viral ribonucleoprotein (vRNP) complexes, which are comprised of viral RNA, nucleoprotein (NP), and the viral polymerase, translocate into the nucleus via the NP nuclear localization signal. Once in the nucleus, IAV genomes are replicated. Thus, NP staining can be used as a surrogate to directly monitor cytosolic-to-nuclear trafficking of incoming IAV particles, prior to the onset of replication. To determine whether MCOLN2 affects entry of IAV, A549 cells ectopically expressing MCOLN2 or an empty vector were infected with IAV in the presence of cycloheximide to inhibit *de novo* protein synthesis, thereby restricting NP detection to incoming virus only. NP localization was monitored over a 3-h time course by immunofluorescence and confocal microscopy (Fig. 4A). We found that compared to control cells, a greater number of MCOLN2-expressing cells contained NP in their nuclei at all time points (Fig. 4B and C). For a control, we also showed that bafilomycin A1, a vacuolar ATPase (vATPase) inhibitor that prevents endosome acidification, blocked NP translocation to the nucleus. To confirm these results, a similar experiment was conducted using nuclear-cytoplasmic fractionation (Fig. 4A). Western blotting revealed that the nuclear fractions of MCOLN2-expressing cells contained significantly more viral NP than control cells did (Fig. 4D and E). Together, these experiments show that MCOLN2 enhances an early step in viral infection prior to replication.

To determine whether MCOLN2 affects later stages of the viral life cycle, we first used a YFV subgenomic reporter replicon that lacks structural proteins essential for virus production and contains a *Renilla* luciferase (Rluc) transgene under control of the viral 5′ untranslated region (5′ UTR) (YFRP-Rluc) (37). This replicon RNA can be used to uncouple viral entry from genome translation and replication by monitoring Rluc levels. YFRP-Rluc RNA synthesized *in vitro* was transfected into *STAT1*^*−/−*^ fibroblasts stably expressing the antiviral transcription factor IRF1 (interferon regulatory factor 1) or MCOLN2. Rluc levels were quantified at early (2 to 6 h) and late time points (24 to 72 h) by luciferase assay to measure viral protein translation and RNA replication, respectively (Fig. 4F). While the antiviral ISG IRF1 significantly inhibited replicon activity, MCOLN2 had no effect at any time point. To confirm this finding, an acid bypass experiment was conducted. A549 cells ectopically expressing MCOLN2 were infected with IAV in the presence of a low-pH buffer. This results in direct fusion of IAV with the plasma membrane, bypassing the normal entry process. Under these conditions, MCOLN2 no longer enhanced viral infection (Fig. 4G). Together, these data suggest that MCOLN2 does not affect postentry stages in the viral life cycle.

### MCOLN2 enhances vesicular trafficking of viruses.

**FIG 5:**
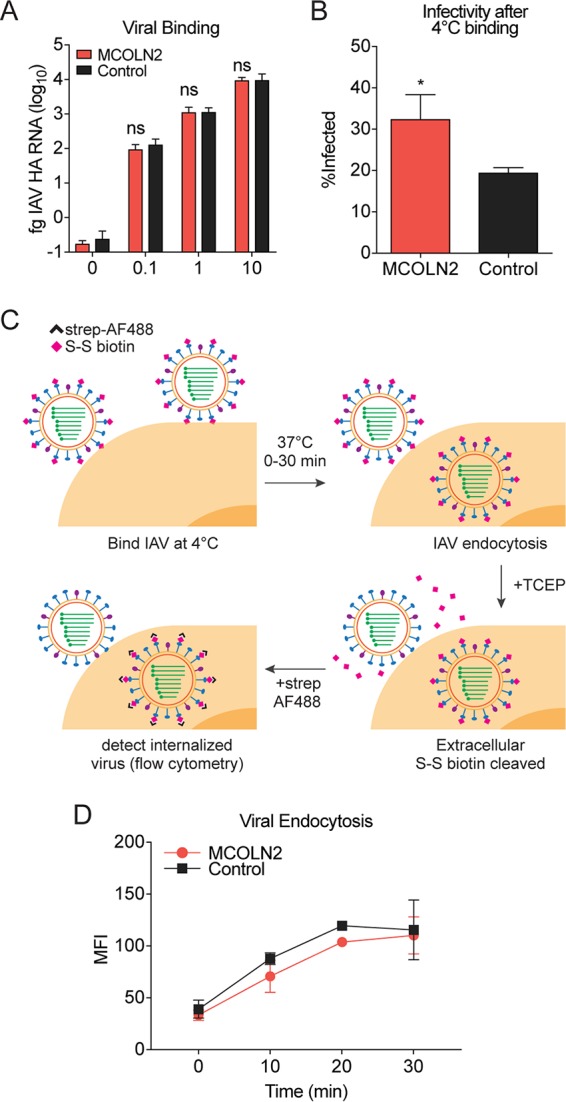
MCOLN2 does not affect cell surface binding or endocytosis of IAV. (A and B) IAV was bound to A549 cells at the indicated MOIs for 1 h at 4°C. The cells were washed and either lysed and cell-bound viral RNA was detected by RT-qPCR analysis (A) or the cells were infected for 8 h at 37°C and subsequently analyzed by staining cells with anti-NP antibody and subsequent flow cytometry-based analysis (B). HA, hemagglutinin. (C) Schematic diagram of the experiment conducted in panel D. (D) Biotinylated IAV (MOI of 10) was bound to A549 cells for 1 h at 4°C. The cells were warmed to 37°C. At the time points shown, cells were treated with 15 mM TCEP, fixed, permeabilized, and stained with streptavidin-AF488. Fluorescence was quantified by flow cytometry. MFI, mean fluorescent intensity. In panels A and B, values are means plus SD from three independent experiments performed in technical triplicate. In panel D, the values are means ± SD (error bars) from two independent experiments performed in technical duplicate. Values that are significantly different (*P* ≤ 0.1) are indicated by an asterisk. Values that are not significantly different (ns) are also indicated.

We next sought to identify which step of viral entry is affected by MCOLN2. To determine whether MCOLN2 modulates attachment of IAV to cells, we bound IAV to control or MCOLN2-expressing A549 cells at 4°C and quantified the amount of cell-bound viral genomic RNA by quantitative RT-PCR (qRT-PCR). At 4°C, IAV can bind to the cell surface but is not efficiently endocytosed into cells. For a control, we shifted a subset of cells to 37°C and quantified infection by flow cytometry. Similar experiments were also conducted with YFV. While MCOLN2 still enhanced infection under the temperature shift conditions, there was no significant difference in the number of viral genomes bound at 4°C to MCOLN2-expressing cells compared to control cells (Fig. 5A and B; also see Fig. S1A in the supplemental material). However, expression of TIM1, a protein known to enhance YFV surface attachment, increased YFV binding to the cells. This indicates that MCOLN2 does not affect attachment of virus to the cell surface.

10.1128/mBio.02314-17.1 FIG S1  (A) YFV-17D Venus was bound to the indicated cell lines at an MOI of 1 for 1 h at 4°C. Cell-bound YFV genomic RNA was quantified by RT-qPCR. (B and C) IAV was labeled with sulfo-NHS-SS-biotin as described in Materials and Methods. (B) Biotinylated or mock-treated IAV titer was determined by plaque assay on MDCK cells. (C) A549 stable cells were infected with biotinylated or mock-treated IAV at an MOI of 0.25 for 8 h. Infection was quantified by staining cells with anti-NP antibody and subsequent flow cytometry-based analysis. Data are presented as the relative infectivity of MCOLN2-expressing cells compared to control cells. (D) Biotinylated IAV was bound to A549 cells at an MOI of 10 for 1 h at 4°C in the presence of EIPA/dynasore or DMSO. Cells were shifted to 37°C. At the indicated time points, cells were treated with 15 mM TCEP or PBS, fixed with 1% PFA, and stained with streptavidin-AF488. Cell fluorescence was quantified by flow cytometry. In panels A to C, data represent means plus SD (error bars) from three independent experiments performed in technical triplicate. In panel D, data represent means plus SD (error bars) from two independent experiments performed in technical duplicate. Values that are significantly different are indicated by asterisks as follows: **, *P* ≤ 0.01; ***, *P* ≤ 0.001. ns, not significantly different. Download FIG S1, PDF file, 0.7 MB. Copyright © 2018 Rinkenberger and Schoggins. 2018 Rinkenberger and Schoggins This content is distributed under the terms of the Creative Commons Attribution 4.0 International license.

To determine whether MCOLN2 affects the rate or total amount of virus endocytosed into cells, we tagged IAV with a sulfo-NHS-SS-biotin (NHS is *N*-hydroxysuccinimide) tag, as previously described (38). The biotin tag allows viral particle detection using streptavidin-conjugated fluorophores. The disulfide bridge linker can be reduced by the cell-impermeable reducing agent Tris(2-carboxyethyl)phosphine (TCEP), allowing efficient tag removal from cell surface-bound IAV, but not from internalized virus. Thus, this assay measures the accumulation of a TCEP-resistant (endocytosed) population of biotin-tagged virus within infected cells over time (Fig. 5C). Biotin accumulation was quantified indirectly by streptavidin-Alexa Fluor 488 staining and subsequent fluorescence detection by flow cytometry.

The method used to tag the virus did not significantly affect the infectivity of the virus (*P* = 0.61 by *t* test) (Fig. S1B). Additionally, MCOLN2 expression still enhanced infection by the labeled virus (Fig. S1C). However, neither the rate nor amount of virus endocytosed into cells expressing MCOLN2 was significantly different from that in control cells. In contrast, treating cells with a combination of 5-(*N*-ethyl-*N*-isopropyl)amiloride (EIPA) and dynasore blocked IAV endocytosis as previously described (Fig. 5D and Fig. S1D) (39). These data suggest that MCOLN2 does not affect endocytosis of viral particles.

**FIG 6:**
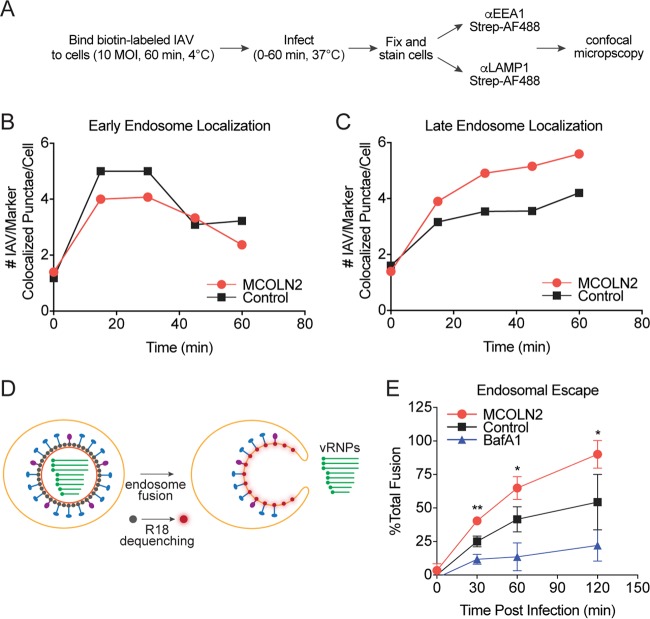
MCOLN2 promotes trafficking of IAV. (A) Schematic diagram of the experiments conducted in panels B and C. (B and C) Biotinylated IAV (MOI of 10) was bound to A549 cells for 1 h at 4°C. The cells were warmed to 37°C for the indicated times. The cells were fixed, treated with 15 mM TCEP, and permeabilized with Triton X-100. The cells were stained with streptavidin-AF488 and anti-EEA1 (B) or anti-LAMP-1 (C) antibodies. IAV-endosome colocalization was determined by confocal microscopy. (D) Illustration of the experiment performed in panel E. (E) R18-labeled IAV (MOI of 10) was bound to A549 cells for 1 h at 4°C. The cells were warmed to 37°C for the indicated times. The cells were fixed, and R18 fluorescence was quantified by flow cytometry. In panels B and C, the results of one representative replicate of three independent experiments are shown. In panel E, values are means ± SD (error bars) from three independent experiments performed in technical duplicate. Values that are significantly different are indicated by asterisks as follows: *, *P* ≤ 0.1; **, *P* ≤ 0.01.

Certain viruses, including IAV and flaviviruses such as dengue virus (DENV) and YFV, require transport from early endosomes to endocytic carrier vesicles (ECVs) or late endosomes for efficient endosomal escape to occur (40–43). To determine whether MCOLN2 promotes the rate or efficiency of this process, we used confocal microscopy to assess IAV colocalization with the early endosome marker EEA1 or the late endosome/lysosome marker LAMP-1 at various time points (Fig. 6A and Fig. S2A). After image acquisition, data files were randomized and deidentified with a computational algorithm, and endosome-IAV colocalization was quantified manually. The number of EEA1- and LAMP-1-staining puncta were not significantly different between control cells and MCOLN2-expressing cells. However, we noted a difference in the number of IAV/EEA1-colocalizing puncta at early time points during entry (*P* = 0.05) (Fig. 6B and Fig. S2B). On average, more virus was present in early endosomes of control cells at early time points than in MCOLN2-expressing cells. This is intriguing considering that similar amounts of IAV are endocytosed (Fig. 5D). In contrast, more virus was present in late endosomes of MCOLN2-expressing cells at later time points (*P* = 0.04) (Fig. 6C and Fig. S3). These data suggest that MCOLN2 promotes the efficiency of IAV trafficking to late endosomes or prevents virion degradation in this compartment, resulting in increased endosomal escape.

10.1128/mBio.02314-17.2 FIG S2  (A) A549 cells were fixed with PFA, permeabilized with 0.2% Triton X-100, and stained with anti-EEA1 or anti-LAMP-1 antibodies followed by BV-421 goat anti-rabbit antibody. The cells were imaged by confocal microscopy. (B) Biotinylated IAV was bound to A549 stable cell lines at an MOI of 10 for 1 h at 4°C. The cells were shifted to 37°C for the time points shown and then treated with 15 mM TCEP or PBS. Samples were prepared as described above, stained with anti-EEA1 antibody, and additionally stained with streptavidin-AF488. The cells were imaged by confocal microscopy. White circles in enlarged insets indicate colocalizing puncta. Download FIG S2, PDF file, 1.3 MB. Copyright © 2018 Rinkenberger and Schoggins. 2018 Rinkenberger and Schoggins This content is distributed under the terms of the Creative Commons Attribution 4.0 International license.

10.1128/mBio.02314-17.3 FIG S3  Biotinylated IAV was bound to A549 stable cell lines at an MOI of 10 for 1 h at 4°C. The cells were shifted to 37°C for time points shown and then treated with 15 mM TCEP or PBS. A549 cells were fixed with PFA, permeabilized with 0.2% Triton X-100, and stained with anti-LAMP-1 antibody followed by BV-421 goat anti-rabbit antibody and streptavidin-AF488. Cells were imaged by confocal microscopy. White circles in enlarged insets indicate colocalizing puncta. Download FIG S3, PDF file, 1.1 MB. Copyright © 2018 Rinkenberger and Schoggins. 2018 Rinkenberger and Schoggins This content is distributed under the terms of the Creative Commons Attribution 4.0 International license.

To assess the effect of MCOLN2 on the degradative capacity of late endosomes/lysosomes, we qualitatively measured late endosomal/lysosomal pH and the ability of MCOLN2-expressing cells to degrade endosomal cargo. To measure endosomal pH, we stained cells with the cell-permeable, pH-sensitive dye acridine orange. No significant difference in staining intensity was observed between control cells and MCOLN2-expressing cells. For a control, we confirmed that the dye was sensitive to ammonium chloride treatment. These data suggest that MCOLN2 does not have a significant effect on the pH of late endosomes or lysosomes (Fig. S4A). To determine the effect of MCOLN2 on the degradative capacity of late endosomes/lysosomes, we quantitated the rate of epidermal growth factor receptor (EGFR) degradation after the addition of its ligand epidermal growth factor (EGF), which is endocytosed and transported to late endosomes/lysosomes where it is degraded by endosomal proteases. MCOLN2-expressing cells degraded EGFR more rapidly than control cells did after EGF addition (Fig. S4B and C), suggesting that MCOLN2 either promotes the transport of EGFR to late endosomes/lysosomes or increases the degradative activity of this compartment. In either case, MCOLN2 does not attenuate the degradative capacity of late endosomes/lysosomes. Together, these experiments further support a model in which MCOLN2 promotes IAV infection by increasing the efficiency of IAV trafficking to late endosomes.

10.1128/mBio.02314-17.4 FIG S4  (A) A549 stable cell lines were incubated in media with 200 mM NH_4_Cl or without NH_4_Cl for 4 h. The cells were subsequently treated with 1 µM acridine orange for 5 min before cell fluorescence was quantitated by flow cytometry. (B and C) Serum-starved A549 stable cell lines were treated with 200 ng/ml EGF or without EGF. At the time points shown, the cells were lysed, and EGFR levels were detected by Western blotting (B) and quantified (C). In panel A, data represent means plus SD (error bars) from three independent experiments performed in technical triplicate. Statistical comparisons were made between treatment conditions and PBS control. In panel C, data represent means ± SD (error bars) from five independent experiments. Values that are significantly different are indicated by asterisks as follows: *, *P* ≤ 0.1; ***, *P* ≤ 0.001; ****, *P* ≤ 0.0001. Download FIG S4, PDF file, 2.1 MB. Copyright © 2018 Rinkenberger and Schoggins. 2018 Rinkenberger and Schoggins This content is distributed under the terms of the Creative Commons Attribution 4.0 International license.

If MCOLN2 enhances viral infection by increasing the efficiency of vesicular trafficking, then we hypothesized that more virus should fuse with and escape from endosomes in cells ectopically expressing MCOLN2. To test this, we took advantage of octadecyl rhodamine B (R18)-labeled IAV. R18 is a lipophilic, self-quenching dye commonly used in the study of enveloped virus fusion kinetics. R18 can be incorporated into the viral envelope at high concentration, resulting in self-quenching of its fluorescence. Fusion of the labeled virus with a host membrane results in dilution of R18 into the host membrane and dequenching of its fluorescent signal, which can be monitored by flow cytometry (Fig. 6D). A549 cells ectopically expressing MCOLN2 or a control vector were infected with R18-labeled IAV. After infection, there was a significant increase in dequenched R18 signal in MCOLN2-expressing cells, indicating that ectopic MCOLN2 expression promotes fusion of IAV (Fig. 6E).

We next wanted to determine whether the role of MCOLN2 in vesicular trafficking could explain why certain seemingly unrelated viruses are affected by MCOLN2. To this end, we reevaluated our previously published screen of 14 viruses and present results here to group viruses based on their MCOLN2 phenotype (2). Viruses were grouped based on membrane fusion/release point: plasma membrane, early endosomes, or endocytic carrier vesicles (ECVs)/late endosomes. Viruses with understudied, disputed, or poorly characterized strain-specific entry mechanisms were left out of this analysis. We then compared these three groups to infection data in MCOLN2-expressing cells (Table 1). We found that only viruses that require transport to ECVs/late endosomes are affected by MCOLN2, supporting our finding that MCOLN2 modulates viral trafficking through the endosomal system.

**TABLE 1:** Virus entry enhanced by MCOLN2

Virus group^a^ and virus	Enhanced?
Plasma membrane	
Coxsackie B virus	No
HIV-1	No
Measles virus	No
Newcastle disease virus	No
Human parainfluenza virus type 3	No
Vaccinia virus	No

Early endosome	
O’nyong nyong virus	No
Respiratory syncytial virus	No

Late endosome/ECV^b^	
Equine arterivirus	Yes
Influenza A virus	Yes
Dengue virus	Yes
Yellow fever virus^b^	Yes

^a^ Viruses were grouped by point of fusion/release into the cell cytoplasm. Viruses were subsequently grouped based on enhancement by MCOLN2, as determined in previous screening studies.

^b^ Viruses that are released from ECVs.

### A rare genetic variant of human MCOLN2 fails to enhance viral infection.

**FIG 7:**
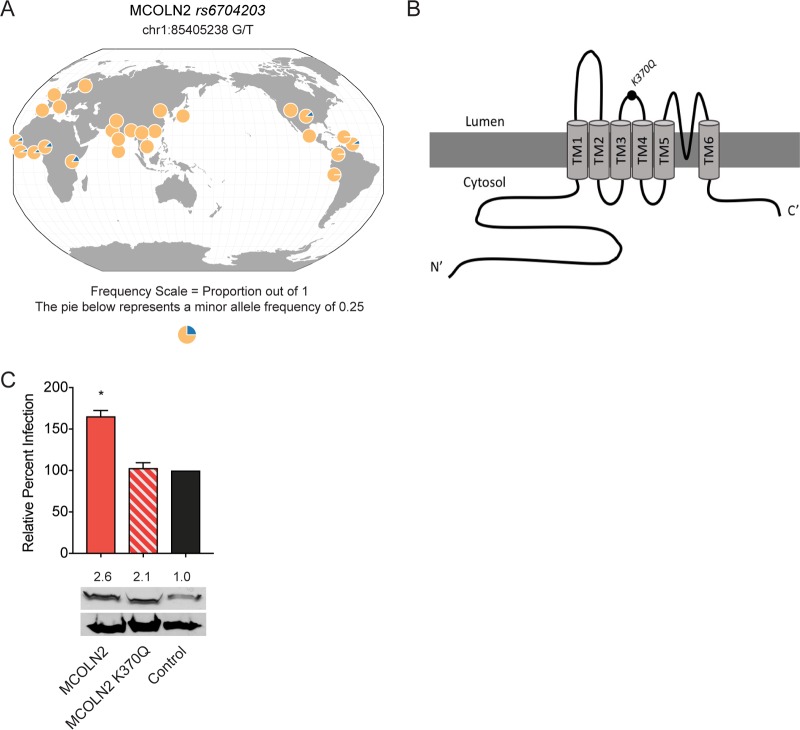
A rare genetic variant of MCOLN2 has a loss of function in viral enhancement. (A) Geographic distribution of *rs6704203*, showing increased frequency of the G allele in African populations. The graphic was generated using GGV browser. (B) Illustration of the location of the K370Q mutation within MCOLN2. Transmembrane domains 1 to 6 (TM1 to TM6) in MCOLN2 are shown. (C, top) A549 cells were infected with IAV (MOI of 0.2) for 8 h. Infection was quantified by staining cells with anti-NP antibody and subsequent flow cytometry-based analysis. (Bottom) MCOLN2 protein expression levels in each cell line were determined by Western blotting. Relative band intensity was quantified by using ImageJ. Values are means plus SD (error bars) from four independent experiments performed in technical triplicate. Values that are significantly different (*P* ≤ 0.1) are indicated by an asterisk.

Loss-of-function mutations in *MCOLN1* cause mucolipidosis type IV disorder (11, 12), while mutations in murine *Mcoln3* cause the Varitint-Waddler phenotype. To date, genetic variation in *MCOLN2* has not been associated with any known disease state. We searched the Phase 3 1000 Genomes database for rare *MCOLN2* nonsynonymous mutations that are predicted to be damaging to the encoded protein (44). We found a G/T single nucleotide polymorphism (SNP) *rs6704203* at chr1:85405238, which encodes a lysine-to-glutamine change at amino acid 370 of MCOLN2. Across 2,504 individuals representing 26 populations worldwide, the genotype frequencies are TT (94.4%, 2,364 individuals), GT (5.3%, 133 individuals), and GG (7 individuals, 0.3%) (Fig. 7A) (45). While the *rs6704203* G allele occurs at a frequency of only 2.9% across all individuals in the 1000 Genomes database, this frequency is enriched to an average of 11% in various African subpopulations. Similar results were obtained from the Exome Aggregation Consortium (EXAC), which contains sequencing data from more than 60,000 individuals (46). Of 121,350 *rs6704203* alleles annotated in the EXAC database, the G allele occurs at a general frequency of 0.77%, with an enrichment to 8.5% in African populations. Only 42 individuals in the EXAC database are homozygous for the *rs6704203* GG genotype, confirming the rareness of this variant. This rare SNP causes a lysine-to-asparagine mutation at residue 370, which is predicted to be located between transmembrane domains 3 and 4 in MCOLN2 (Fig. 7B). To test whether MCOLN2-K370Q affects the viral enhancing phenotype in our heterologous system, we first expressed the mutant and monitored expression levels by Western blotting. Both wild-type MCOLN2 and MCOLN2-K370Q were expressed at similar levels, indicating that the mutation does not disrupt expression (Fig. 7C). A549 cells ectopically expressing MCOLN2-K370Q showed a near complete loss of viral enhancement compared to the wild type (Fig. 7C). These data indicate that K370Q disrupts the ability of MCOLN2 to enhance viral infection, raising the intriguing possibility that humans bearing one or two G alleles at *rs6704203* may have altered susceptibility to certain viral infections.

## DISCUSSION

The first challenge a virus must overcome during infection is gaining access to the host cell cytoplasm. To this end, viruses have developed ways of exploiting host factors and existing uptake pathways in order to efficiently enter cells. Significant progress has been made in identifying the mechanisms and cellular factors necessary for viral entry. Viruses often use attachment factors such as heparin sulfate, sialic acid, and gangliosides to bind to the cell surface and subsequently associate with one or more receptors such as DC-SIGN (dendritic cell-specific intercellular adhesion molecule 3-grabbing nonintegrin), decay-accelerating factor (DAF), coxsackievirus and adenovirus receptor (CAR), and integrins. This results in either direct penetration of the virion into the cytoplasm or receptor-mediated endocytosis which can involve macropinocytosis, phagocytosis, clathrin-mediated endocytosis, caveolar endocytosis, or other pathways (1). Many endocytosed viruses are quickly delivered to and escape from Rab5-positive early endosomes which can be promoted by acidic endosomal pH as well as endosomal cathepsins and furin-like proteases. However, some viruses such as IAV and flaviviruses delay membrane fusion until after trafficking from early to late endosomes (47). Our study identifies MCOLN2 as a novel endosomal host factor that facilitates viral vesicular trafficking to ultimately promote productive infection. Specifically, we show that MCOLN2 promotes the efficiency with which certain viruses are transported from early to late endosomal compartments.

TRP superfamily proteins are commonly associated with sensory signaling processes. The TRPML/MCOLN subfamily seems to depart from this paradigm, having reported functions in basic cellular processes, including lysosome fusion events, recycling, endocytosis, and autophagy (17–21, 23, 25, 27, 28, 30, 48). Recently, there has been increasing evidence for MCOLN family proteins playing a role in immune function and bacterial infection (2, 3, 29, 31, 34). In this study, we found evidence for a direct role for MCOLN2 in the context of viral entry, further expanding the functional repertoire of this protein family. An intriguing finding from our study comes from the endocytosis (Fig. 5D), endocytic trafficking (Fig. 6B and C), and membrane fusion (Fig. 6E) experiments. We note that the endocytosis experiment discriminates between the total amount of cell-bound and internalized virus, whereas the endocytic trafficking experiments quantitate specific subsets of the internalized virus population based on colocalization. These data indicate that while the same amount of virus is endocytosed into MCOLN2-overexpressing cells compared to control cells, more virus in the MCOLN2-overexpressing cells successfully traffics to strongly acidified endosomal compartments, from which it is subsequently released. In contrast, at early time points in control cells, the virus appears to be delayed in the early endosome compartment (Fig. 6B). This finding suggests that a population of IAV is normally lost or diverted in control cells during the entry process but is better retained in a productive endocytic pathway in MCOLN2-expressing cells. Additional studies are needed to determine the fate of this IAV population in control cells.

MCOLN2 has been reported to be induced by LPS and type I IFN (31, 33), hence its inclusion in our prior ISG screens (2, 3). We have also found MCOLN2 to be induced by type I IFN and poly(I⋅C) treatment (Fig. 1A). To our knowledge, this is the first interferon-inducible factor reported to affect viral vesicular trafficking. It is therefore interesting to consider the implications of MCOLN2 upregulation during viral infection *in vivo*, when IFN responses are systemic. In nonimmune cells, basal or IFN-induced MCOLN2 expression may lead to enhanced viral uptake in a manner that benefits the virus, i.e., by viral hijacking. However, in immune cells, which express higher levels of basal MCOLN2 (31, 32, 34), increased viral uptake could result in increased PAMP recognition, a stronger immune response, and subsequently improved viral clearance, which benefits the host. The latter model is supported by our finding that MCOLN2 expression results in increased ISG expression after SINV infection (Fig. 2B). Deciphering these models will be important for determining whether MCOLN2-specific agonists or antagonists could be used therapeutically to modulate viral infection.

Last, we found that a rare genetic variant of human *MCOLN2* fails to enhance viral infection when ectopically expressed in cell culture. Intriguingly, this allelic variant is found at higher frequencies in African populations compared to all geographic populations. Additional studies are needed to characterize the nature of the K370Q mutation encoded by *rs6704203*. Moreover, our current data on this variant are only correlative. To determine whether humans with one or two copies of the rare allele have altered susceptibility to viral infection, in-depth genetic and clinical studies from multiple individuals or families would be needed.

## MATERIALS AND METHODS

### Cell lines and viruses.

A549 lung cancer, U-2 OS osteosarcoma, and human embryonic kidney-derived 293T cells were grown in Dulbecco modified Eagle medium (DMEM) supplemented with 10% fetal bovine serum (FBS) and 0.1 mM nonessential amino acids. *STAT1*^*−/−*^ fibroblasts and THP-1 cells were grown in RPMI 1640 medium supplemented with 10% FBS and 0.1 mM nonessential amino acids. Stable cell lines transduced with SCRPSY lentiviral vectors (49) were selected for 3 days in media supplemented with 4 µg/ml puromycin and maintained in normal media.

The construction, characterization, and generation of viral stocks for the following viruses have been previously described: EAV-GFP (EAV labeled with green fluorescent protein [GFP]) (derived from infectious clone pEAV211-GFP2aT [26]), SINV-A-GFP (derived from infectious clones pS300-GFP [27]), VEEV-GFP (derived from infectious clone pTC83-GFP [4]). YFV-17D-Venus was produced by electroporation of *STAT1*^*−/−*^ fibroblasts with *in vitro*-transcribed RNA as previously described (3). Influenza A virus (IAV) (strain A/WSN/33) was propagated in MDCK cells as previously described (50). For entry assays, IAV was concentrated by pelleting through a 30% sucrose cushion in phosphate-buffered saline (PBS) with Ca^2+^/Mg^2+^ using ultracentrifugation in an SW-28 rotor at 110,000 × *g* for 1 h.

### Plasmids and cloning.

MCOLN2, IRF1, and control constructs were prepared as previously described (3). Plasmids containing MCOLN1 or MCOLN3 open reading frames were kindly provided by Neal Alto. TIM-1 (also known as HAVCR1) was obtained from the DNASU Plasmid Repository. pENTR.MCOLN2 was used as a starting point for all mutagenesis. MCOLN2 D463D/KK and K370Q mutants were generated from wild-type MCOLN2 by overlap extension PCR using primer sets listed in Table S1 in the supplemental material. All genes were amplified out of their respective plasmids by PCR and cloned into pDONR.221 using BP Clonase (Invitrogen) according to the manufacturer’s protocol. Genes were cloned into the previously described lentiviral, puromycin-selectable, red fluorescent protein (RFP)-coexpressing, SCRPSY-DEST vector (49) using LR Clonase II (Invitrogen) per the manufacturer’s protocol. For CRISPR/Cas9 experiments, MCOLN2 and control targeting guides were cloned into plentiCRISPRv2 (a gift from Feng Zhang, Addgene plasmid 52961) (Table S1) (51).

10.1128/mBio.02314-17.5 TABLE S1  List of primer sets used during cloning and PCR-based methods. Download TABLE S1, PDF file, 0.02 MB. Copyright © 2018 Rinkenberger and Schoggins. 2018 Rinkenberger and Schoggins This content is distributed under the terms of the Creative Commons Attribution 4.0 International license.

### Lentivirus production and viral infections.

SCRPSY lentiviruses were produced as previously described (49). For lentiCRISPRv2 production, 293T cells were seeded at 4 × 10^5^ cells per well into six-well plates. The next day, cells were transfected with 1 µg of lentiCRISPRv2, 0.2 µg of plasmid expressing VSV G protein (VSVg), and 0.8 µg of plasmid expressing HIV-1 gag-pol using X-tremeGENE 9 (Roche). The medium was changed 6 h later, and lentivirus-containing culture supernatants were collected at 48 and 72 h posttransfection. Pooled supernatants were clarified by centrifugation at 800 × *g* for 5 min. Polybrene and HEPES were added to a final concentration of 4 µg/ml and 25 mM, respectively. Lentivirus was stored at −80°C until use.

For lentivirus transductions, cells were seeded at 7 × 10^4^ cells per well in 24-well plates. The next day, the medium was changed to DMEM supplemented with 4 µg/ml Polybrene, 3% FBS, and 25 mM HEPES. Cells were transduced by spinoculation at 800 × *g* for 45 min at 37°C. The medium was changed 6 h later to DMEM supplemented with 10% FBS and 0.1 mM nonessential amino acids, and cells were replated 48 h after transduction for subsequent experimentation.

Cells were seeded at 1 × 10^5^ in 24-well plates 24 h prior to infection. For IAV infections, cells were infected with virus diluted in 200 µl DMEM containing 0.1% FBS and 0.3% bovine serum albumin (BSA) for 1 h at 37°C. For all other infections, cells were infected with virus diluted in 200 µl DMEM containing 1% FBS for 1 h at 37°C. The virus inoculum was removed and replaced with 1 ml normal growth medium. At the designated time, cells were dislodged with Accumax, centrifuged at 800 × *g* for 2 min at 4°C, fixed in 1% paraformaldehyde (PFA) for 10 min, and resuspended in 1× PBS with 3% FBS for flow cytometry analysis.

### Viral binding assay.

A549 cells were seeded at 1 × 10^5^ cells per well in 24-well plates one day prior to infection. The cells were chilled at 4°C for 30 min, followed by virus binding at 4°C in DMEM containing 0.1% FBS and 0.3% BSA for 1 h. The cells were washed twice with chilled 1× PBS. The cells were either lysed in RLT buffer, and RNA was isolated using an RNeasy minikit (Qiagen) or shifted to 37°C in DMEM containing 10% FBS for 8 h and harvested for quantitation of infection by flow cytometry. Viral RNA in isolated RNA samples was quantified by reverse transcription-quantitative PCR (RT-qPCR) using primers listed in Table S1.

### Acid bypass.

A549 cells were seeded at 1 × 10^5^ cells in 24-well plates. The next day, the cells were chilled at 4°C for 30 min. IAV (multiplicity of infection [MOI] of 10) was bound to cells at 4°C in DMEM containing 0.1% FBS and 0.3% BSA for 1 h. The medium was then changed to DMEM (pH 5, 50 mM acetic acid) prewarmed to 37°C for 5 min. Afterward, the medium was changed to DMEM (pH 7) supplemented with 0.1% FBS, 0.3% BSA, and 2 µg/ml tosylsulfonyl phenylalanyl chloromethyl ketone (TPCK)-treated trypsin (Sigma) for 8 h. The cells were dislodged with Accumax, fixed, and stained for infection quantitation by flow cytometry as described above.

### Nuclear NP localization.

For image-based analysis, A549 cells were seeded at 2 × 10^4^ cells in eight-well chamber slides. The next day, cells were prechilled on ice for 30 min and incubated with IAV (MOI of 10) for 1 h at 4°C in 150 µl DMEM containing 0.1% FBS and 0.3% BSA. Three hundred microliters of prewarmed DMEM (containing 10% FBS and 0.15 mM cycloheximide) with or without 15 nM bafilomycin A1 was added to each well, and the slides were incubated at 37°C. At the indicated time points, wells were incubated in PBS with 4% PFA and Ca^2+^/Mg^2+^ for 10 min and left in PBS with Ca^2+^/Mg^2+^ overnight before immunofluorescence. Samples were imaged using a Zeiss LSM 780 confocal microscope. Images were quantified using ImageJ software.

For cell fractionation-based analysis, A549 cells were seeded at 1 × 10^7^ cells in p150 dishes. The next day, the cells were chilled to 4°C for 30 min and infected with IAV (MOI of 10) for 1 h at 4°C in 2 ml DMEM containing 0.1% FBS and 0.3% BSA. The medium was changed to 10 ml prewarmed DMEM (containing 10% FBS and 0.15 mM cycloheximide) with or without 15 nM bafilomycin A1 (warmed). The cells were scraped from plates in 5 ml cold PBS with Ca^2+^/Mg^2+^ at the indicated time points. The cells were washed three times in chilled PBS with Ca^2+^/Mg^2+^ and centrifuged at 200 × *g* for 3 min. The cell pellets were resuspended in 500 µl cold double-distilled water (ddH_2_O) for 10 min and lysed with 10 to 20 strokes of a type B Dounce homogenizer. Lysis efficiency was checked by phase-contrast microscopy. Nuclei were pelleted at 200 × *g* for 10 min and stored at −80°C. The cytoplasm-containing supernatant fraction was centrifuged at 1,500 × *g* for 10 min to remove the remaining nuclei and stored at −80°C.

### Immunofluorescence.

Wash buffer (WB) consisted of PBS with Ca^2+^/Mg^2+^ supplemented with 1% BSA and 0.1% Tween 20. The cell samples were permeabilized for 10 min with 0.2% Triton X-100 in WB and blocked for 1 h in WB. The samples were incubated with the following antibodies or label in WB for 2 h: anti-nucleoprotein (anti-NP) antibody (Sigma) diluted 1:1,000, anti-EEA1 (Abcam) diluted 1:500, anti-LAMP-1 (Abcam) diluted 1:500, and/or streptavidin-Alexa Fluor 488 (AF488) (Abcam) diluted 1:500. The samples were then washed three times in WB for 1 min each time and probed with goat anti-mouse antibody conjugated to Alexa Fluor 488 (Life Technologies) diluted 1:1,000 or goat anti-rabbit antibody conjugated to BV421 (BD Biosciences) diluted 1:200 in WB for 1 h. The samples were washed twice with WB, the nuclei were stained for 5 min with Hoechst 33342 (Life Technologies) in WB, and the samples were mounted with Prolong Gold Antifade reagent (Thermo Fisher).

### Plaque assays.

*STAT1*^*−/−*^ fibroblasts and A549 cells were seeded at 7 × 10^4^ cells per well in 24-well plates for YFV-17D and IAV infections, respectively. The cells were infected, and supernatants were harvested at 8 h (IAV) or 24 h (YFV). For YFV, supernatants were serially diluted and used to infect BHK-21J cells. The plates were overlaid with DMEM supplemented with 0.1% NaHCO_3_, 10 mM HEPES, 4% FBS, and 1.2% Avicel. Four days later, the cells on the plates were fixed with 3.7% formaldehyde, and plaques were visualized with crystal violet staining. For IAV, collected supernatant was serially diluted and used to infect MDCK cells seeded at 1 × 10^6^ in six-well plates the previous day. The plates were overlaid with DMEM supplemented with 0.1% NaHCO_3_, 0.2% BSA, 1.2% Avicel, and 10 µg/ml TPCK. Two days later, the cells on the plates were fixed with 3.7% formaldehyde, and plaques were visualized with crystal violet staining.

### Internalization assay.

Concentrated IAV stocks were diluted to 1 mg/ml viral protein and labeled with sulfo-NHS-SS-biotin (Thermo Fisher) at 65 nM for 2 h at room temperature. The reaction was quenched by adding glycine (pH 7) to 0.1 M. Labeled virus was purified by ultracentrifugation through a 30% sucrose cushion in an SW-28 rotor at 110,000 × *g* for 1 h. Labeling efficiency was determined using a Pierce biotin quantitation kit per the manufacturer’s instructions. A549 cells were washed in PBS, treated with 0.05% trypsin, centrifuged at 200 × *g* for 2 min, and resuspended in 200 µl DMEM containing 0.1% FBS and 0.3% BSA at 50,000 cells per well in a 96-well plate. Control samples were incubated in medium containing 400 µM EIPA and 80 µM dynasore or 5% DMSO for 30 min at 37°C. Cells were pelleted by centrifugation at 200 × *g* for 2 min, and IAV (MOI of 20) was bound to cells at 4°C in 50 µl chilled DMEM containing 0.1% FBS and 0.3% BSA for 1 h. The cells were centrifuged at 200 × *g* for 2 min, resuspended in 50 µl DMEM containing 0.1% FBS, 0.3% BSA, 400 µM EIPA, and 80 µM dynasore or 5% DMSO prewarmed to 37°C. At the indicated time points, 150 µl chilled 15 mM Tris(2-carboxyethyl)phosphine (TCEP) was added for 5 min, the cells were centrifuged at 200 × *g* for 2 min, and the cell pellets were resuspended in chilled 4% PFA. The cells were permeabilized with 0.5% saponin, stained for 30 min with 1 µg/ml streptavidin-conjugated Alexa Fluor 488, and fluorescence intensity was quantified by flow cytometry.

### Vesicular trafficking assay.

IAV was biotinylated as described above. A549 cells were plated at a density of 1 × 10^4^ in eight-well chamber slides. The next day, the slides were chilled for 30 min on ice. Virus was bound to cells at an MOI of 10 in 150 µl DMEM containing 0.1% FBS and 0.3% BSA at 4°C for 1 h. The medium was changed to prewarmed DMEM containing 0.1% FBS, 0.3% BSA, and 0.15 mM cycloheximide and incubated at 37°C. At the indicated time points, the cells were treated with 15 mM TCEP for 5 min, fixed in 4% PFA for 10 min, permeabilized with 0.2% Triton X-100, and costained with streptavidin-conjugated Alexa Fluor 488 and either anti-EEA1 or anti-LAMP-1 (Abcam) antibodies as described above. Images were taken with a Zeiss LSM 780 confocal microscope. After image acquisition, data files were randomized and deidentified with a computational algorithm. Streptavidin-endosomal marker colocalization was manually counted for at least 10 cells per condition.

### Endosomal escape assay.

IAV was labeled similarly to the protocol previously described (52). Briefly, concentrated IAV was diluted to 100 µg/ml viral protein. Six microliters of 1.2 mM rhodamine B in ethanol (EtOH) was added per ml of IAV for 1 h at room temperature. The labeling reaction was filtered through a 0.22-µm filter, and virus was purified on a 30 to 50% sucrose gradient in an SW-40 rotor at 220,000 × *g* for 90 min. A549 cells were washed in PBS, treated with 0.05% trypsin, and centrifuged at 200 × *g* for 2 min. The cell pellets were resuspended in 200 µl DMEM containing 0.1% FBS and 0.3% BSA at 50,000 cells per well in a 96-well plate. The cells were chilled to 4°C for 30 min. Labeled IAV was bound to cells at an MOI of 1 in 200 µl DMEM containing 0.1% FBS and 0.3% BSA for 1 h at 4°C. The cells were centrifuged at 200 × *g* for 2 min, and the cell pellets were resuspended in 150 µl DMEM containing 0.1% FBS and 0.3% BSA warmed to 37°C. At the indicated time points, 50 µl of 4% PFA was added to each well. The cells were resuspended in PBS containing 3% FBS, and fluorescence intensity was quantified by flow cytometry.

### Western blotting.

Samples were run on a 10% polyacrylamide SDS-polyacrylamide gel, transferred to a nitrocellulose membrane, and blocked for 30 min with 5% milk in Tris-buffered saline containing 0.1% Tween 20 (0.1% Tween 20 TBS-T). The membranes were probed with one of the following primary antibodies: anti-actin (Abcam) diluted 1:3,000, anti-NP (Millipore) diluted 1:1,000, anti-lamin B (Santa Cruz) diluted 1:1,000, or anti-MCOLN2 (Origene) diluted 1:200. For standard experiments, the membranes were washed with 0.1% Tween 20 TBS-T and probed with goat anti-rabbit or goat anti-mouse antibodies conjugated to horseradish peroxidase (HRP) (Pierce). The membranes were washed with 0.1% Tween 20 TBS-T, incubated with enhanced chemiluminescence (ECL) substrate (Pierce) according to the manufacturer’s instructions, and exposed to film. For quantitative experiments, membranes were probed with goat anti-mouse or donkey anti-goat antibodies conjugated to IRdye infrared dye (Li-Cor). The membranes were washed with Tris-buffered saline (TBS), and signal was detected using a Li-Cor Odyssey system.

### Endosomal pH measurement.

A549 cells were treated with 50 mM NH_4_Cl or without NH_4_Cl for 4 h. A549 cells were detached with Accumax and resuspended at 1 × 10^5^ cells per condition. Acridine orange was added to a final concentration of 1 µg/ml with or without 50 mM NH_4_Cl to cell suspensions for 5 min before cell fluorescence was quantitated with flow cytometry.

### EGFR degradation assay.

A549 cells were plated in 24-well plates at 1 × 10^5^ cells per well with standard growth media. The cells were washed two times with PBS, and the medium was changed to DMEM without serum for 4 h. The medium was changed to DMEM containing 0.15 mM cycloheximide with 200 ng/ml EGF or without EGF. At 0, 0.5, 1, 2, or 3 h later, the cells were washed with PBS and lysed in radioimmunoprecipitation assay (RIPA) buffer. EGFR levels relative to actin were quantitated by Western blotting as described above.

### Antibody staining and flow cytometry.

For quantitation of IAV infections, infected cells were permeabilized and stained with anti-NP HT103 diluted 1:1,000 using the Cytofix/Cytoperm kit according to the manufacturer’s instructions (BD Bioscience). HT103 was kindly provided by Thomas Moran. Samples were subsequently stained with goat anti-mouse conjugated to AF488 (Invitrogen), and cell fluorescence was quantified by flow cytometry. An S1000 flow cytometer (Stratedigm) was used, and data were quantified using FlowJo. On average, 20,000 cells were counted per condition. A minimum of 10,000 cells were counted per condition. For the internalization assay specifically, a minimum of 2,000 cells was counted per condition due to cell loss.

### RT-qPCR.

RNA was isolated using an RNeasy minikit (Qiagen) according to the manufacturer’s instructions. For reverse transcription-quantitative PCR (RT-qPCR), the Quantifast SYBR green PCR kit (Qiagen) was used according to the manufacturer’s instructions. RNA concentration was determined by using a NanoDrop spectrophotometer, and 40 ng total RNA was added to each reaction mixture along with one of the following QuantiTect primer sets (Qiagen): QT00090895 for MCOLN2, QT00090895 for Mx1, QT000203763 for IFN-β, QT00236824 for Arf6, QT00212730 for CC chemokine ligand 2 (CCL2), QT02289294 for IFIT2, QT00099274 for IFI27, or QT00061516 for RPS11. The reaction mixtures were analyzed in a 7500 fast real-time PCR thermal cycler (Applied Biosystems). For analysis, expression of the housekeeping gene RPS11 was used for sample normalization.

### Statistical analysis.

Statistical significance for most data sets was determined using a Student’s *t* test. For normalized data sets, a ratio paired *t* test was used. A single outlier from each data set, if present, was identified using Dixon’s Q test and removed.
