# Supplementary figures and images for: Mucolipin-2 Cation Channel Increases Trafficking Efficiency of Endocytosed Viruses
Source: mBio. 2018 Jan 30;9(1):e02314-17. doi: 10.1128/mBio.02314-17 (PMC5790917; doi:10.1128/mBio.02314-17)

Figure S1

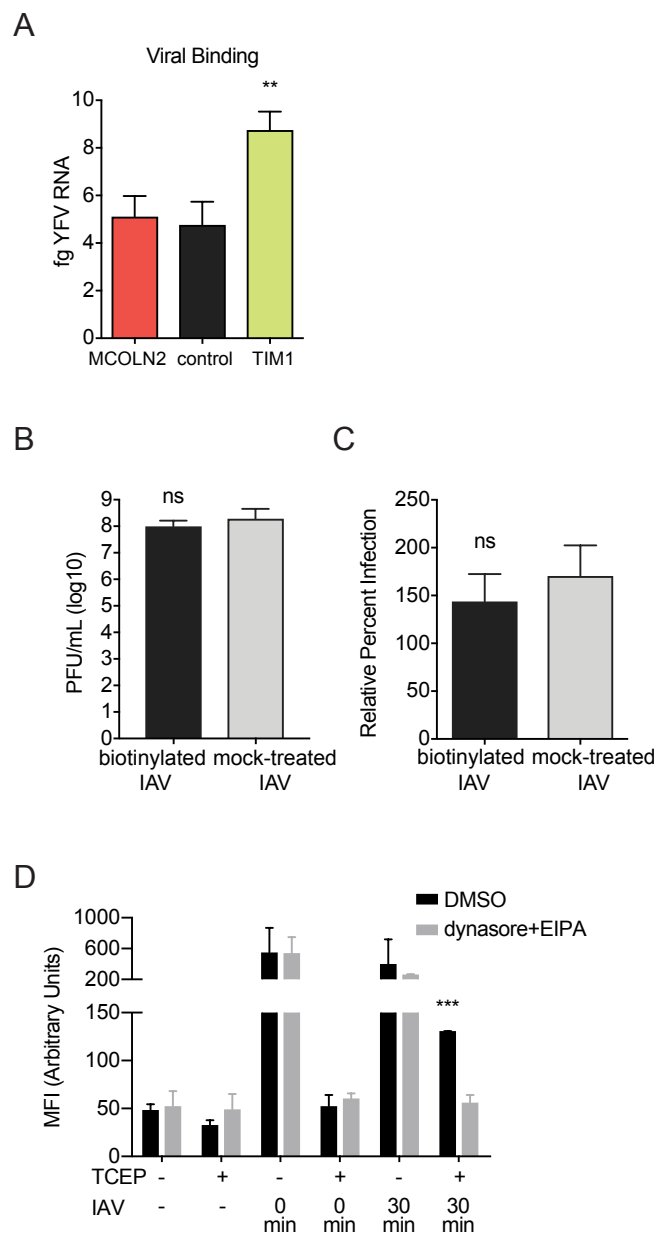

Supplement: FIG S1 [file mbo001183701sf1.pdf]

Figure S2

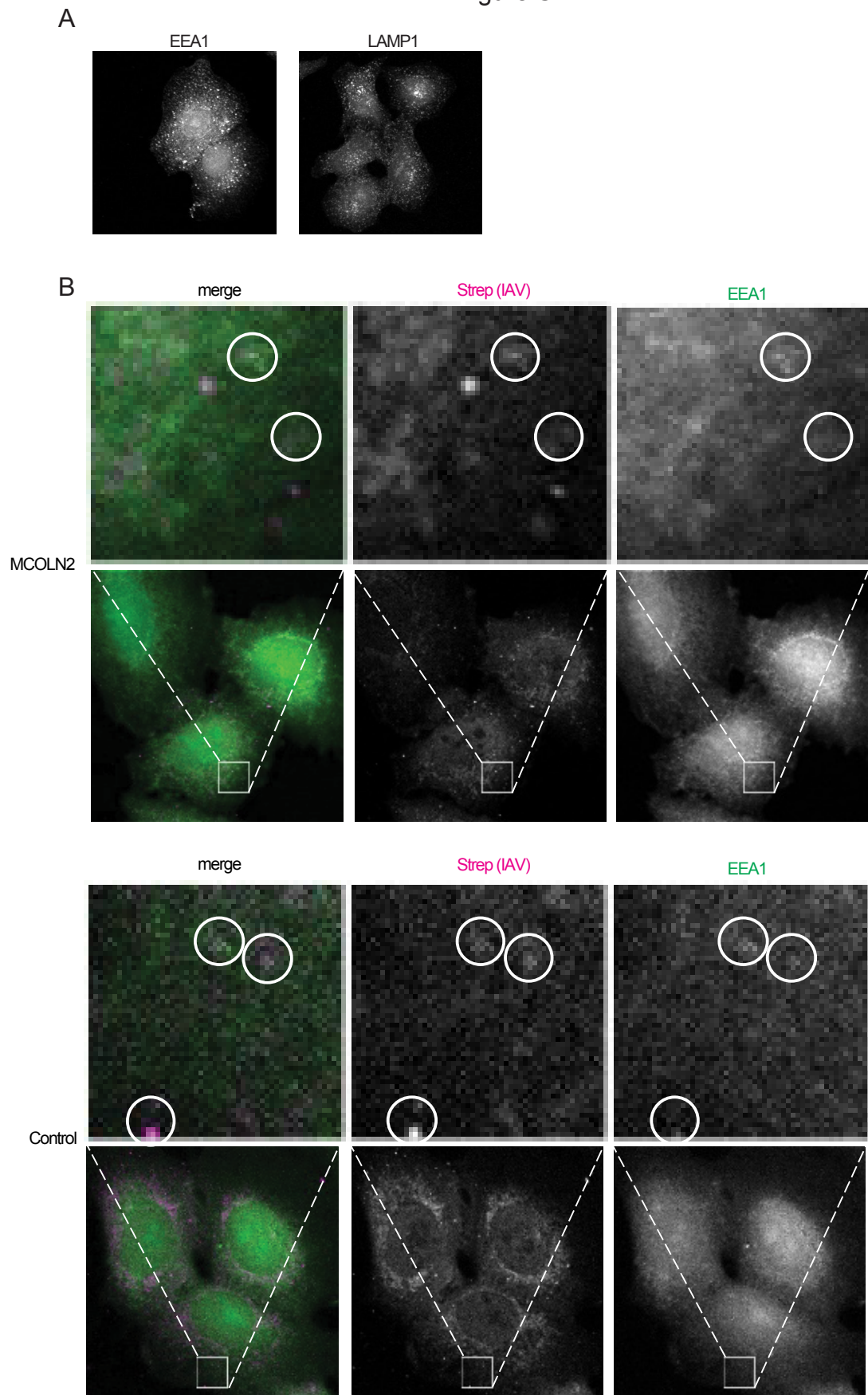

Supplement: FIG S2 [file mbo001183701sf2.pdf]

Figure S3

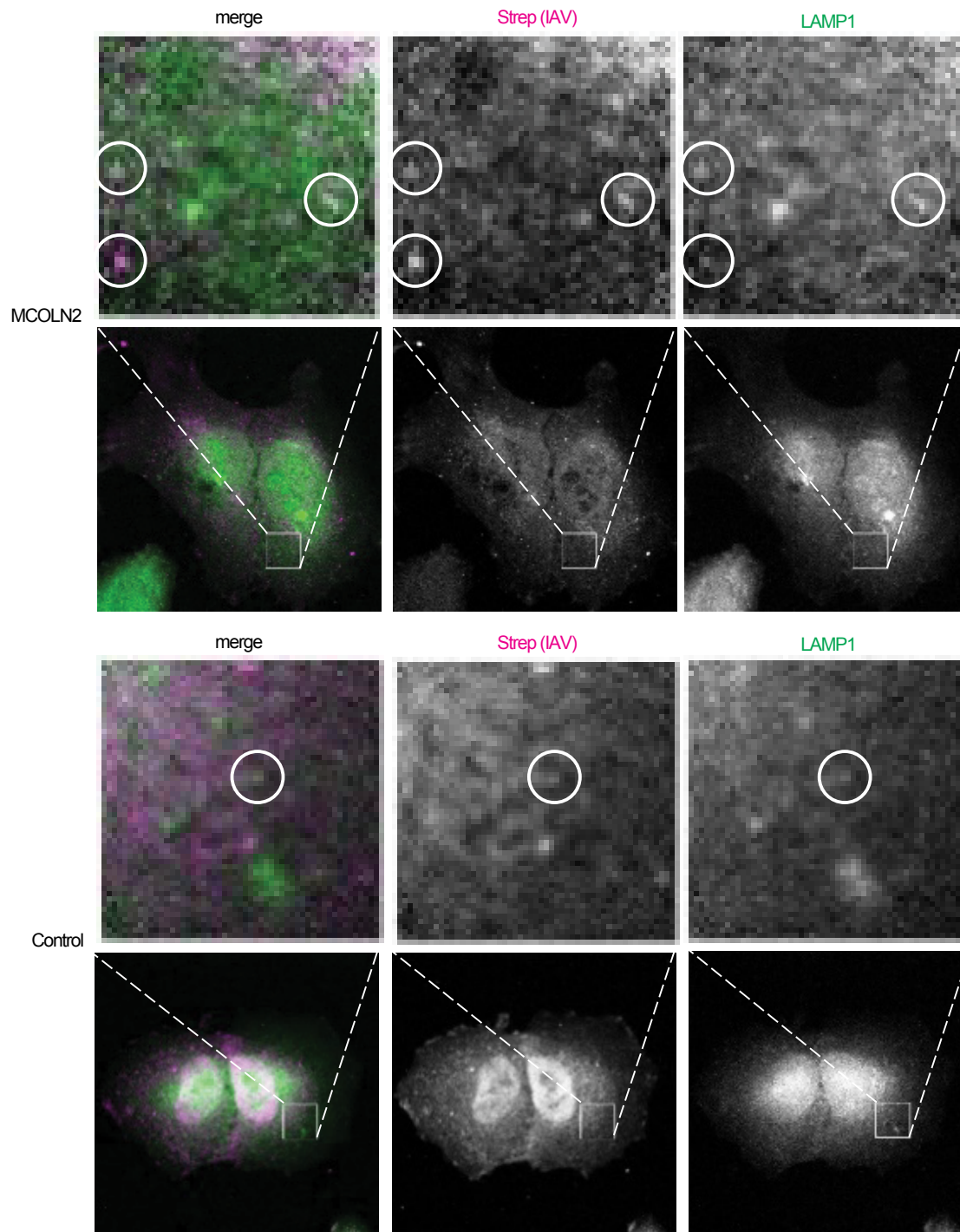

Supplement: FIG S3 [file mbo001183701sf3.pdf]

Figure S4

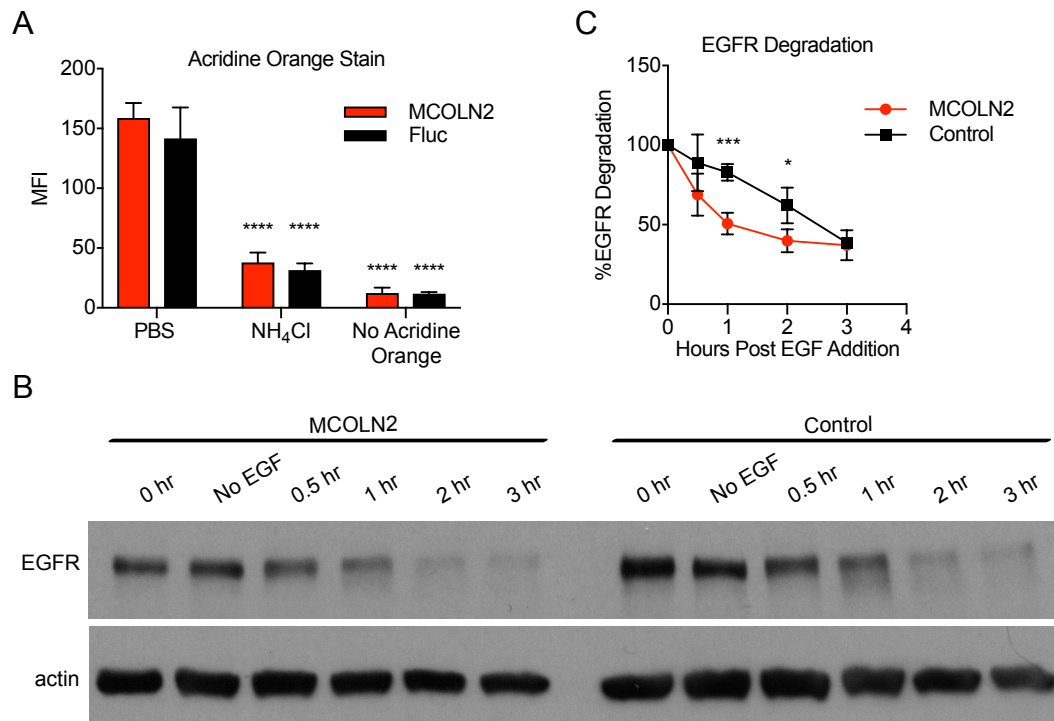

Supplement: FIG S4 [file mbo001183701sf4.pdf]
