# Supplementary material for: Mucolipin-2 Cation Channel Increases Trafficking Efficiency of Endocytosed Viruses
Source: mBio. 2018 Jan 30;9(1):e02314-17. doi: 10.1128/mBio.02314-17 (PMC5790917; doi:10.1128/mBio.02314-17)
Supplement: TABLE S1 [file mbo001183701st1.pdf]

Table S1 : Primer sequences used in this study

Name	Forward Primer	Reverse Primer
**MCOLN2 BP Cloning**	5'-ggggacaagtttgtacaaaaaagcaggcttcaccatggcacatcgtgattctgagatgaa-3'	5'-ggggaccactttgtacaagaaagctgggtttagctaataggtatcaagtgatcatcact-3'
**MCOLN2 D463D/KK Mutagenesis**	5'-ctggatttgggcaaaggttgcaaacatcttcttaccgttgaccagagaaaacagacac-3'	5'-gtgtctgttttctctggtcaacggtaagaagatgtttgcaacctttgcccaaatccag-3'
**MCOLN2 K370Q Mutagenesis**	5'-aatggaaatcaaagcaaataatctcacaaactatgatctctgc-3'	5'-gcagagatcatagtttgtgagattatttgctttgatttccatt-3'
**sgMCOLN2 #1**	5'-caccgaacccagtttccacggaatc-3'	5'-cgattccgtggaaactgggttcaaa-3'
**sgMCOLN2 #2**	5'-caccgcagctaaaggacattaccct-3'	5'-cagggtaatgtcctttagctgcaaa-3'
**sgMCOLN2 #3**	5'-caccgtctccaagaagcctccggac-3'	5'-cgtccggaggcttcttggagacaaa-3'
**sgControl**	5'-caccggttggcatattggcccagac-3'	5'-aaacgtctgggccaatatgccaacc-3'
**A/WSN/33 HA – RT-qPCR**	5'-taacctgctcgaagacagac-3'	5'-agagccatccggtgatgtta-3'
**YFV-17D – RT-qPCR**	5'-aatcgagttgctaggcaataaacac-3'	5'-tccctgagctttacgaccaga-3'
